# The complexities of bacterial-fungal interactions in the mammalian gastrointestinal tract

**DOI:** 10.15698/mic2016.05.497

**Published:** 2016-03-16

**Authors:** Eduardo Lopez-Medina, Andrew Y. Koh

**Affiliations:** 1 Department of Pediatrics, Universidad del Valle and Centro de Estudios en Infectología Pediátrica, Cali, Colombia.; 2 Departments of Pediatrics and Microbiology, University of Texas Southwestern Medical Center, Dallas, Texas, USA

**Keywords:** autophagy, trypanosome, cell death, differentiation, proliferation, starvation

The mammalian gastrointestinal (GI) tract is host to trillions of microorganisms (including bacteria, fungi, and viruses), some of which can cause invasive and potentially life-threatening infections when the host’s immune defenses are compromised. Cancer and stem cell transplant patients are particularly vulnerable to microorganisms translocating from the gut, with gram-negative bacteria such as *Pseudomonas aeruginosa* and the most common human fungal pathogen *Candida albicans* having significant medical impact. While much has been learned by studying fungal or bacterial pathogens in isolation, the interactions of bacteria and fungi living within the mammalian host are not well understood and may have significant consequences in the pathogenesis of infectious diseases. Here, we showcase two studies that explore bacterial-fungal interactions that modulate infectious disease pathogenesis: one in which commensal bacteria mediate host suppression of *C. albicans* gut colonization; the other in which *C. albicans* mediates suppression of *P. aeruginosa* virulence in the gut. Understanding how microbes interact and antagonize each other in specific body niches may help us identify new potential therapeutic targets for preventing or treating microbial infections.

With advances in medicine, particularly in cancer therapy and transplantation medicine, a burgeoning population of immunocompromised patients has emerged. These patients are highly vulnerable to infections with opportunistic pathogens. As a result, the use of prophylactic or empiric antibiotics to prevent bacterial, fungal, and viral infections is the standard of care. As a consequence of increased antibiotic exposure, the development of microbial antibiotic resistance is rising, and the unintended deleterious effect of antibiotics on resident microbiota is starting to be appreciated.

An imbalance in gut microbiota populations, also known as dysbiosis, can be precipitated by host genetics, dietary changes, and most notably exposure to antibiotics. There is a growing body of evidence that gut dysbiosis can be associated with a number of human pathogenic states. For instance, in cancer and stem cell transplant patients, endogenous bacteria and fungi can translocate from the gut and cause life-threatening bloodstream infections. Thus, when gram-negative bacteria (e.g. Enterobacteriaceae such as *E. coli* or *Enterococcal* spp.) burden increases in the gut, the risk of invasive bacteremia with these pathogens significantly increases 1.

Commensal anaerobic bacteria in the gut provide a key defense mechanism by inhibiting the growth of potentially pathogenic bacteria. One possible mechanism for maintaining pathogen GI colonization resistance involves commensal anaerobe induction of mucosal immune effectors (e.g. antimicrobial peptides, AMP) that kill the pathogen. Manipulation of these mucosal immune effectors can not only decrease GI colonization with potentially pathogenic bacteria but also decrease the risk of invasive infection 2.

Gut commensal anaerobic bacteria can also modulate commensal fungi gut colonization 3,4. Commensal fungi, notably *Candida* spp, colonize the GI tracts of numerous mammals, including humans. In hosts with a competent immune system, colonization with *Candida* spp. does not result in disease or infection. But severely immunocompromised patients (cancer, transplant, neonatal and intensive care unit patients) are at high risk for developing invasive *Candida* infection, many of which are thought to originate from the gut.

Since colonization is a prerequisite for both bacterial and fungal invasive disease, studying the interactions of gut bacterial and fungi and understanding how these interactions affect not only colonization but invasive infectious disease pathogenesis will be critical for developing novel methods to prevent these infections. With this thought in mind, our research group focused on the long-standing observation that adult mice are resistant to GI colonization by *C. albicans* (CA), the most common human fungal pathogen. Germ-free mice and neonatal mice (with immature gut microbiota), however, can be sustainably colonized with CA. Furthermore, CA colonization resistance in mice is abrogated by antibiotic treatment, but only antibiotics most effective in depleting (killing) anaerobic bacteria resulted in the highest CA colonization levels. These data suggest that a mature and intact gut microbiota, particularly commensal anaerobes, are critical for maintaining CA resistance in mice 3.

Gut microbiota profiling (using both 16S rRNA sequencing and bacterial group specific qPCR) of CA colonization-resistant (no antibiotic treatment or antibiotics that did not deplete anaerobes) or CA colonization-susceptible (treated with anti-anaerobic antibiotics) revealed that Bacteroidetes and clostridial Firmicutes were the most effective in promoting CA colonization resistance. Not surprisingly, endogenous gut fungi levels also increase in mice treated with anti-anaerobic antibiotics. Antibiotic exposure in humans has long been associated with increased risk of superficial fungal or yeast infections (i.e. oral thrush, yeast vaginitis, or yeast diaper rash). When antibiotic-treated or germ-free CA-colonized mice are orally gavaged with a representative Bacteroidetes (*Bacteroides thetaiotamicron*) or clostridial Firmicute (*Blautia producta*), CA is eliminated from the GI tract, whereas other representatives of the gut microbiota, including other commensal anaerobes, fail to promote CA gut colonization resistance. What is most striking, however, is that two Bacteroidetes species belonging to the same genus could have divergent effects on CA colonization: *B. theta* promoting CA reduction while *Bacteroides fragilis* having no appreciable affect on CA colonization 3. Thus, microbial species-specific effects can drive distinct host responses and/or result in unique microbiota phenotypes 5.

The mechanistic basis for commensal anaerobic induction of CA resistance appears to rely on the induction of gut-derived host immune effectors, similar to how commensal anaerobes maintain Enterobacteriaceae or Enterococcal colonization resistance (Figure 1A). Both *B. theta* and *B. producta* induce greater colonic expression of the transcription factor HIF-1a, an important regulator of mammalian innate immunity, and the cathelicidin antimicrobial peptide LL-37 (CAMP, the mouse ortholog) with anti-*Candida* activity, as compared with other commensal bacteria. In addition to microbial induced indirect host effects, both *B. theta* and *B. producta* produce small-chain fatty acids (SCFAs) that can induce host immune responses, including induction of Treg cells 6 and AMPs 7 (Figure 1C). Furthermore, SCFAs at physiologically relevant doses directly inhibits CA growth *in vitro* and decreases CA colonization in mice, suggesting that bacterially produced SCFAs may play a critical role in CA resistance 3 (Figure 1B).

**Figure 1:**
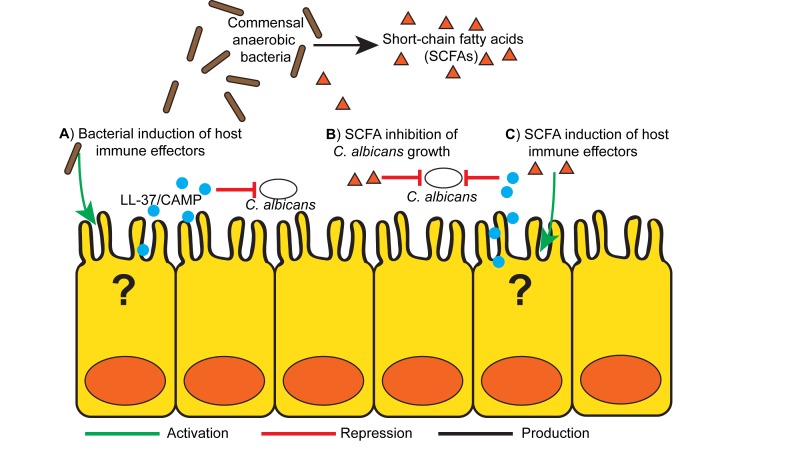
Figure 1: Proposed mechanism of gut bacteria mediated *Candida albicans* gastrointestinal colonization resistance. **(A)** Commensal gut bacteria, particularly obligate anaerobes (i.e. *Bacteriodetes thetaiotamicron*, *Blautia producta*), induce production of colonic host immune effectors, specifically antimicrobial peptides such as LL-37/CAMP which have Candicidal activity. **(B)** Bacterially derived fermentation products, short-chain fatty acids (SCFAs) may have a direct effect on *C. albicans* growth and colonization. (**C**) SCFAs can induce production of colonic antimicrobial peptides, including cathelicidins (LL-37/CAMP) and defensins that have activity against *C. albicans*.

Pharmacologic activation of HIF-1A using the Koa hoale seed derivative L-mimosine induces LL-37 production and ultimately reduces CA levels in an *in vitro* fungicidal assay using co-cultured human colonocytes, and this effect is nullified by *HIF1A* knockdown. When applying a similar strategy *in vivo*, CA GI colonization levels significantly decreased in wild-type mice treated with mimosine but not in mimosine-treated mice with Hif1a deleted from the intestinal epithelium. Strikingly, the mimosine-induced CA colonization reduction translated to a 50% decrease in mortality from CA invasive disease. Similar approaches of HIF1a activation of myeloid cells 8,9 and keratinocytes 10 have been used to combat acute bacterial infection. Using *Camp* knockout mice, CAMP was also necessary for the mimosine-induced antifungal effects 3.

While HIF-1α and CAMP are required for the commensal anaerobe-induced production against CA colonization in antibiotic treated mice, this was not true in the absence of antibiotics. It may be that with an intact and mature gut microbiota, the loss of one immune pathway (e.g. HIF/CAMP) is easily compensated for by other redundant immune effectors (e.g. other AMPs such as defensins) that also may maintain CA colonization resistance. In the setting of a markedly stressed and imbalanced gut microbiota (e.g. after antibiotics), the gut microbiota (input) stimulus may be so diminished that the loss of one immune pathway cannot be compensated for.

While mice are CA colonization resistant, reportedly up to 80% of human are colonized with CA 11. How is this discrepancy explained? Estimates of high CA human colonization rates were based on studies conducted in western societies. More recent studies of humans living in remote and "traditional or primitive" societies exhibit a CA GI carriage rate of less than 10% 12,13, whereas other *Candida* spp. are more prevalent, consistent with the mycobiota of other mammals including rodents. Therefore, CA may not be an expected or normal member of the commensal gut microbiota in humans, but perhaps a more recently acquired "commensal" resulting from advances in human technology (i.e. treatment antibiotics, antibiotics in the food chain, and adoption of diets with greater refined carbohydrate and fat content) that induce gut microbiota changes which promote CA colonization. We have recently identified a human CA stool strain that is able to colonize the GI tract of mice with intact gut microbiota, and this clinical isolate is genetically distinct from the CA strains we used in the study described above (unpublished observation, AYK). 

In summary, anaerobic commensal bacteria, particularly Bacteroidetes and clostridial Firmicutes clusters IV and XIVa, prevent or reduce GI *C. albicans* colonization in the murine model through activation of HIF1A and LL-37. In these severely immunocompromised cancer or stem cell transplant patients, augmenting innate cellular function or mucosal integrity is virtually impossible, so maintaining an intact gut microbiota and thus boosting GI mucosal immune defenses to reduce GI colonization of potentially pathogenic pathogens (such as CA) may be a novel method for preventing infections in these high-risk patients. 

While the previous study 3 highlighted the importance of gut commensal bacteria inhibiting gut fungi, the corollary (fungi inhibiting or antagonizing a bacterium) is obviously possible. Therefore, we also chose to study the interactions of CA and the bacterium *P. aeruginosa* (PA): two pathogens that often inhabit the same body niches (gut, lung, burn wounds) and can cause devastating "opportunistic" infections in the immunocompromised host. The gut was used as the body niche of interest, since PA has a long and storied past of causing invasive bloodstream infections in cancer patients and thought to originate from the gut 14. Despite being ubiquitous in the environment, PA is not a normal member of the human gut microbiota. Gut microbiota homeostasis (and thus pathogen colonization resistance) is a primary defense mechanism to prevent bacterial translocation from the gut. So in order to overcome the gut’s inherent resistance to PA colonization, antibiotics are utilized to deplete commensal anaerobes in the mouse gut and thus promote PA (and CA) gut colonization 15. 

Interestingly, while CA does not affect PA’s ability to colonize the murine GI tract, the presence of CA prevents PA dissemination in the setting of neutropenia. Intact cellular immunity, particularly neutrophils, is another key defense mechanism against bacterial translocation from the gut and is sufficient for PA dissemination (but not for CA dissemination) 4,15. CA inhibition of PA dissemination is not strain dependent and holds true when testing three PA and four CA strains (in all combinations) 16. 

*In vivo* PA transcriptome analysis (using RNA-Seq) reveals CA-induced suppression of PA pyochelin and pyoverdine biosynthetic pathways. Pyochelin and pyoverdine play a critical role in iron acquisition and virulence. So one possible explanation as to why CA would suppress PA pyochelin and pyoverdine expression is to provide a competitive advantage for iron. Increasing iron concentrations can also inhibit PA pyochelin and pyoverdine gene expression, but the presence of CA does not increase overall iron levels in the mouse gut. Given the suppression of iron acquisition via pyochelin/pyoverdine, PA could be expected to have difficulty growing and thus lower GI colonization levels. But deletion of pyochelin and pyoverdine has no effect on PA GI colonization. As a testament to the importance of iron, PA (and other microorganisms) utilizes additional strategies to acquire iron (e.g. FeoABC system in PA), thus may allow sustained growth and colonization of the GI tract (Figure 2A). 

**Figure 2:**
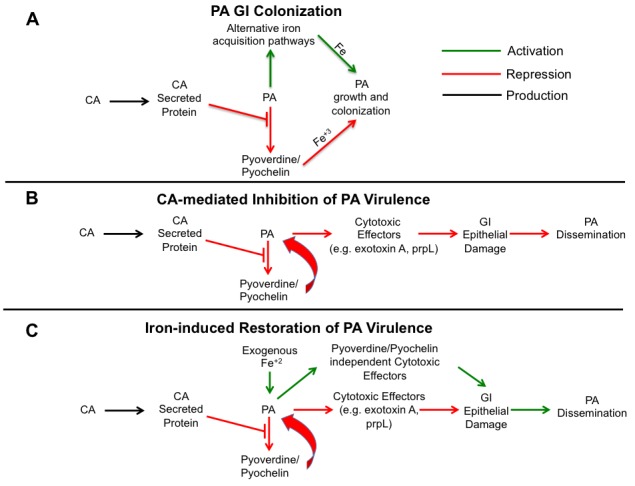
Figure 2. Proposed mechanism of *Candida *albicans (CA) inhibition of *Pseudomonas aeruginosa *(PA) virulence in the gut. **(A) PA gastrointestinal (GI) colonization. **Despite inhibition of pyoverdine and pyochelin gene expression by CA, PA is able to colonize the murine GI tract, perhaps by utilizing alternative iron acquisition pathways (e.g. FeoABC system) that allow sustained growth and colonization of the gut. **(B) CA-mediated Inhibition of PA Virulence. **CA inhibits PA pyochelin and pyoverdine expression, most likely through a secreted protein. Production of PA extracellular virulence effectors, such as PrpL and exotoxin A, are decreased. Host gut epithelial integrity remains intact, and PA dissemination is prevented **(C) Iron-induced restoration of PA virulence. **Iron supplementation in PA/CA co-colonized mice induces pyochelin-pyoverdine independent PA cytotoxic effector molecular production leading to increased gut permeability and mucosal barrier damage. PA can now disseminate from the gut.

Deleting pyochelin or pyoverdine genes, however, significantly decreases the mortality from *P. aeruginosa* dissemination. Interestingly, pyoverdine is not only a siderophore important for iron acquisition but also a signaling molecule that induces the production of two extracellular virulence factors, the protease PrpL and exotoxin A 17. Since an intact intestinal mucosal barrier is another primary defense mechanism preventing microbial translocation from the GI tract in humans and mice 4,15,18, we postulated that CA-mediated protection from PA invasive infection might be due to inhibition of PA extracellular cytotoxic molecule production (i.e. exotoxin A, PrpL, etc). Indeed, CA (more specifically CA-secreted proteins) inhibits the production of PA extracellular cytotoxic molecules, such as exotoxin A, and thus inhibits the cytotoxic effect on cultured colonocytes 16. These findings are consistent with our previous work demonstrating that disruption of PA cytotoxic virulence effector genes (e.g. Type III secretion system *ExoU*) also results in loss of cytotoxicity and significantly reduces virulence in a PA gut model without affecting PA GI colonization 19 (Figure 2B).

Moreover, CA-secreted factors (most likely extracellular proteins) inhibit PA pyochelin/pyoverdine gene expression and pyoverdine production *in vitro*. Interestingly, administration of heat-killed CA is unable to suppress PA virulence in mice, but CA culture supernatants and supernatant proteins significantly decrease PA virulence 16. 

Finally, iron and iron-overload states have been shown to increase the virulence of bacteria 20, and consistent with these observations, iron supplementation restores PA virulence in PA-CA co-colonized mice. Iron supplementation does not promote PA expansion (increased burden) in the gut and likely has a negligible inhibitory effect on host phagocytosis (since the mice are profoundly neutropenic already). In fact, iron supplementation restores PA virulence by modulating gut epithelial integrity (increasing gut permeability) indirectly through PA and not directly via the host (Figure 2C). Hence, in contrast to the first study in which CA colonization modulation via host immune effectors ultimately results in decreased virulence, we describe a novel observation of a fungal-inhibition of bacterial effectors critical for virulence but not important for colonization.

In both studies by Fan, *et al* 3 and Lopez-Medina, *et al* 16, we utilize an “artificial” but now all too common niche, the antibiotic-treated gut with concomitant pathogenic microbe (PA or CA) expansion, to gain insight in bacterial-fungal interactions in clinically common scenarios: immunocompromised (neutropenia ¬with or without gut epithelial disruption) hosts with antibiotic induced gut dysbiosis. Specifically, we describe two examples where bacterial-fungal interactions results in attenuation of invasive infectious disease: 1) commensal bacteria-mediated host suppression of CA colonization and 2) CA-mediated suppression of PA virulence. Thus, further studies of the synergistic or antagonistic bacterial-fungal interactions in the GI tract may allow us to identify novel therapeutic methods (i.e CA-secreted proteins or gastrointestinal mucosal immune effectors) to prevent or control difficult to treat infections, an increasingly important clinical challenge given the rising tide of bacterial and fungal antibiotic resistance.

## References

[1] Taur Y, Xavier JB, Lipuma L, Ubeda C, Goldberg J, Gobourne A, Lee YJ, Dubin KA, Socci ND, Viale A, Perales MA, Jenq RR, van den Brink MR, Pamer EG (2012). Intestinal domination and the risk of bacteremia in patients
undergoing allogeneic hematopoietic stem cell
transplantation.. Clin Infect Dis.

[2] Brandl K, Plitas G, Mihu CN, Ubeda C, Jia T, Fleisher M, Schnabl B, DeMatteo RP, Pamer EG (2008). Vancomycin-resistant enterococci exploit antibiotic-induced
innate immune deficits.. Nature.

[3] Fan D, Coughlin LA, Neubauer MM, Kim J, Kim MS, Zhan X, Simms-Waldrip TR, Xie Y, Hooper LV, Koh AY (2015). Activation of HIF-1alpha and LL-37 by commensal bacteria inhibits
Candida albicans colonization.. Nat.

[4] Koh AY, Kohler JR, Coggshall KT, Van Rooijen N, Pier GB (2008). Mucosal damage and neutropenia are required for Candida albicans
dissemination.. PLoS Pathog.

[5] Buffie CG, Bucci V, Stein RR, McKenney PT, Ling L, Gobourne A, No D, Liu H, Kinnebrew M, Viale A, Littmann E, van den Brink MR, Jenq RR, Taur Y, Sander C, Cross JR, Toussaint NC, Xavier JB, Pamer EG (2015). Precision microbiome reconstitution restores bile acid mediated
resistance to Clostridium difficile.. Nature.

[6] Smith PM, Howitt MR, Panikov N, Michaud M, Gallini CA, Bohlooly YM, Glickman JN, Garrett WS (2013). The microbial metabolites, short-chain fatty acids, regulate
colonic Treg cell homeostasis.. Science.

[7] Schauber J, Svanholm C, Termen S, Iffland K, Menzel T, Scheppach W, Melcher R, Agerberth B, Luhrs H, Gudmundsson GH (2003). Expression of the cathelicidin LL-37 is modulated by short chain
fatty acids in colonocytes: relevance of signalling
pathways.. Gut.

[8] Peyssonnaux C, Datta V, Cramer T, Doedens A, Theodorakis EA, Gallo RL, Hurtado-Ziola N, Nizet V, Johnson RS (2005). HIF-1alpha expression regulates the bactericidal capacity of
phagocytes.. The Journal of clinical investigation.

[9] Zinkernagel AS, Peyssonnaux C, Johnson RS, Nizet V (2008). Pharmacologic augmentation of hypoxia-inducible factor-1alpha
with mimosine boosts the bactericidal capacity of
phagocytes.. J Infect Dis.

[10] Nizet V, Ohtake T, Lauth X, Trowbridge J, Rudisill J, Dorschner RA, Pestonjamasp V, Piraino J, Huttner K, Gallo RL (2001). Innate antimicrobial peptide protects the skin from invasive
bacterial infection.. Nature.

[11] Bougnoux ME, Diogo D, Francois N, Sendid B, Veirmeire S, Colombel JF, Bouchier C, Van Kruiningen H, d'Enfert C, Poulain D (2006). Multilocus sequence typing reveals intrafamilial transmission and
microevolutions of Candida albicans isolates from the human digestive
tract.. Journal of clinical microbiology.

[12] Angebault C, Djossou F, Abelanet S, Permal E, Ben Soltana M, Diancourt L, Bouchier C, Woerther PL, Catzeflis F, Andremont A, d'Enfert C, Bougnoux ME (2013). Candida albicans is not always the preferential yeast colonizing
humans: a study in Wayampi Amerindians.. J Infect Dis.

[13] Xu J, Mitchell TG (2003). Geographical differences in human oral yeast
flora.. Clin Infect Dis.

[14] Tancrede CH, Andremont AO (1985). Bacterial translocation and gram-negative bacteremia in patients
with hematological malignancies.. J Infect Dis.

[15] Koh AY, Priebe GP, Pier GB (2005). Virulence of Pseudomonas aeruginosa in a murine model of
gastrointestinal colonization and dissemination in
neutropenia.. Infect Immun.

[16] Lopez-Medina E, Fan D, Coughlin LA, Ho EX, Lamont IL, Reimmann C, Hooper LV, Koh AY (2015). Candida albicans Inhibits Pseudomonas aeruginosa Virulence
through Suppression of Pyochelin and Pyoverdine
Biosynthesis.. PLoS Pathog.

[17] Lamont IL, Beare PA, Ochsner U, Vasil AI, Vasil ML (2002). Siderophore-mediated signaling regulates virulence factor
production in Pseudomonasaeruginosa.. Proc Natl Acad Sci U S A.

[18] Berg RD (1999). Bacterial translocation from the gastrointestinal
tract.. Adv Exp Med Biol.

[19] Koh AY, Mikkelsen PJ, Smith RS, Coggshall KT, Kamei A, Givskov M, Lory S, Pier GB (2010). Utility of in vivo transcription profiling for identifying
Pseudomonas aeruginosa genes needed for gastrointestinal colonization and
dissemination.. PLoS One.

[20] Litwin CM, Calderwood SB (1993). Role of iron in regulation of virulence genes.. Clinical microbiology reviews.

